# Association between type of feeding at discharge from the hospital and nutritional status of very low birth weight preterm infants

**DOI:** 10.1590/1414-431X20176540

**Published:** 2018-03-01

**Authors:** F.P. Martins-Celini, W.A. Gonçalves-Ferri, D.C. Aragon, J.P. Bernichi, C. Calixto, E.M.F. Sacramento, M.A. Santos, F.E. Martinez

**Affiliations:** Departamento de Puericultura e Pediatria, Faculdade de Medicina de Ribeirão Preto, Universidade de São Paulo, Ribeirão Preto, SP, Brasil

**Keywords:** Very low birth weight, Nutrition, Feeding, Nutritional status, Human milk

## Abstract

The ideal feeding for premature babies has been the source of extensive debate. The aim of this study was to assess the association between type of feeding at discharge and the nutritional status of very low birth weight infants. This was a retrospective cohort of preterm babies with birth weight ≤1500 g, born between January 2006 and December 2013. The infants were divided into 3 groups according to type of feeding at discharge: exclusive breast milk (group 1), mixed feeding (group 2) and exclusive artificial formula (group 3). Frequencies of each group were calculated, as well as mean Z-score differences in weight, length and head circumference. Six hundred and forty-nine newborns were included. The mean weight of groups 1, 2, and 3 was 1338.7, 1104.0, and 1254.7 g, respectively, and their mean gestational age was 31.9, 30, and 31.2 weeks, respectively. The Z-score differences (means±SD) for groups 1, 2, and 3 were: −0.84±0.68, −1.02±0.75, and −0.86±0.71 for weight, −0.21±1.23, −0.52±1.64 and −0.08±1.34 for head circumference, and −1.10±1.18, −1.54±1.37, and −0.97±1.21 for length. A significant difference was observed between groups 2 and 3 in the adjusted Z-score model for length, with no significant differences in anthropometric measurements for the other comparative analyses. Because of its many advantages, breastfeeding should be stimulated within neonatal units since nutritional status was not influenced by the different types of feeding.

## Introduction

The feeding of preterm newborns, especially those weighing less than 1500 g at birth, has been a source of increasing concern. There has been debate about the ideal type of feeding that would allow an adequate development for these babies after birth, with growth and weight gain rates close to those observed during intrauterine life (1).

Despite the efforts to offer adequate nutritional support, postnatal growth restriction is still frequently observed in neonatal intensive care units (2). Postnatal factors causing clinical instability may contribute to this outcome (3).

Human milk is recommended for the enteral feeding of premature and term newborns since it is better tolerated, and is associated with low rates of necrotizing enterocolitis, sepsis, retinopathy of prematurity, and a better neurocognitive development (4–8). Human milk is known to contain many antioxidant substances that are important for the reduction of oxidative stress (7).

Infant milk formulas are used in situations in which the milk of the mother or of other donors is not available. Some neonatal units use these formulas to improve the weight gain of very low birth weight newborns (9,10). The prevention of growth restriction of preterm newborns during hospitalization is of extreme importance since there is evidence suggesting that appropriate postnatal growth is associated with better neurological development and other benefits such as improved immune response and reduced risk of infections (11,12).

In preterm infants, inadequate nutrition in the first months of life can persist throughout childhood, adolescence, and adulthood, and lead to precocious puberty and obesity (13).

The objective of the present study was to determine the association between type of feeding at discharge and the nutritional status of very low birth weight infants.

## Material and Methods

We selected patients included in the Brazilian Neonatal Network of infants with birth weight ≤1500 g, born from January 2006 to December 2013 at a university hospital. Exclusion criteria were: newborns who died, babies with malformations, babies transferred to other institutions, and babies whose medical charts were incomplete.

We calculated differences of Z-scores for weight, length and head circumference measured at hospital discharge and at birth for each patient (Δ= hospital discharge - birth). Simple and multiple linear regression models were adjusted to compare the three groups in terms of Δ Z-score for the anthropometric measurements. For all multiple models, we considered covariates such as bronchopulmonary dysplasia, peri-intraventricular hemorrhage, SNAPPE II (Score for Neonatal Acute Physiology with Perinatal extension-II), weight and gestational age at birth, occurrence of necrotizing enterocolitis and sepsis. Estimates of the differences among group means and their respective 95% confidence intervals were obtained using the SAS 9.3 software (SAS Institute Inc., USA).

The patients included in the study were divided into 3 groups. Group 1 consisted of infants who were exclusively breastfed at discharge, group 2 infants received mixed feeding, i.e., breast milk complemented with infant milk formula, and group 3 consisted of patients exclusively receiving a formula.

In our service, all preterm infants start to be fed milk expressed from their own mother unprocessed or pasteurized breast milk from the milk bank. When a volume of 100 mL·kg^-1^·day^-1^ is reached, the milk is enriched with a fortifying agent. When the patient is clinically stable and receiving a full diet (140–160 mL·kg^-1^·day^-1^), and if the maintenance of maternal lactation is not possible, a full or partial transition to infant milk formulas is started.

According to the nutritional routine of the hospital service, group 1 infants received a greater proportion of human milk, frequently fortified, while group 2 infants received breast milk at least once a day associated with milk formula. Group 3 infants received fortified human milk at the beginning of life, followed by transition to an artificial formula due to the impossibility of maintaining maternal lactation.

For the study, we assumed that the patients who were discharged while being exclusively breastfed received a greater quantity of human milk during hospitalization than patients who were receiving mixed feeding or artificial feeding at discharge.

The study was approved by the Research Ethics Committee (protocol No. 1.018.827) and all mothers gave written informed consent to participate.

## Results

**Figure 1.**
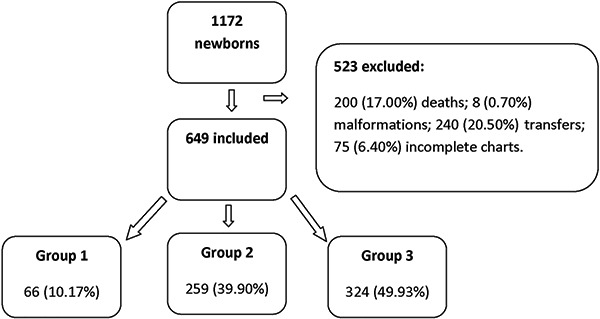
Flowchart of participant selection according to type of feeding at discharge: exclusive breast milk (Group 1), mixed feeding (Group 2) and exclusive artificial formula (Group 3).

A total of 1172 patients were first included. Of these, 523 were later excluded, as shown in Figure 1, and 649 newborns completed the study.

The mean birth weight was 1338.7 g for group 1, 1104.0 g for group 2 and 1254.7 g for group 3. Mean gestational age at birth was 31.9, 30.0 and 31.2 weeks, respectively, and mean corrected age at discharge was 37.8, 41.7 and 38.2 weeks, respectively. The groups were comparable, with no significant difference between them.

The characteristics of the mothers and comorbidities of the newborns included in the study are described in Table 1.

**Table 1.** Characteristics of the mothers and comorbidities of the newborns according to type of feeding at discharge.

	Group 1	Group 2	Group 3
**Comorbidities**			
BPD			
Yes	6 (3.11)	128 (66.32)	59 (30.57)
PIVH			
Grade 1 and 2	9 (6.72)	66 (49.25)	59 (44.03)
Grade 3 and 4	3 (5.77)	34 (65.38)	15 (28.85)
Apgar score			
≤5	2 (5.56)	23 (63.89)	11 (30.56)
>5	64 (10.56)	231 (38.12)	311 (51.32)
SNAPPE			
<20	56 (13.18)	140 (32.94)	229 (53.88)
≥20	10 (4.65)	117 (54.42)	88 (40.93)
Gender			
Male	32 (9.28)	143 (41.45)	170 (49.28)
Female	33 (10.96)	115 (38.21)	153 (50.83)
Late onset sepsis			
Yes	12 (4.41)	148 (54.41)	112 (41.18)
Necrotizing enterocolitis			
Yes	2 (3.77)	30 (56.60)	21 (39.63)
**Maternal characteristics**			
Age			
<20 years	7 (5.69)	56 (45.53)	60 (48.78)
≥20 years	58 (11.09)	201 (38.43)	264 (50.48)
Schooling			
<10 years	15 (6.85)	96 (43.84)	108 (49.32)
>10 years	50 (12.38)	149 (36.88)	205 (50.74)
Chorioamnionitis			
Yes	3 (3.39)	30 (50.85)	27 (45.76)
Antenatal steroid			
No	18 (7.03)	111 (43.36)	127 (49.61)
Delivery			
Vaginal	21 (9.86)	94 (44.13)	98 (46.01)
Cesarean	45 (10.37)	163 (37.56)	226 (52.07)

Data are reported as numbers (%). Study groups: 1, exclusive breast milk; 2, mixed feeding; 3: exclusive artificial formula. BPD: bronchopulmonary dysplasia; PIVH: periintraventricular hemorrhage; SNAPPE-II (Score for Neonatal Acute Physiology with Perinatal extension-II).

Table 2 lists the Z-scores for weight, length, and head circumference of each group.

**Table 2.** Differences in Z-scores for weight, length, and head circumference between discharge and birth according to type of feeding at discharge.

Study groups	Weight	Length	Head circumference
1	−0.84 (0.68)	−1.10 (1.18)	−0.21 (1.23)
2	−1.02 (0.75)	−1.54 (1.37)	−0.52 (1.64)
3	−0.86 (0.71)	−0.97 (1.21)	−0.08 (1.24)

Data are reported as ΔZ-Score [mean differences in Z-scores (SD)] for weight, length and skull perimeter between discharge and birth. Study groups: 1, exclusive breast milk; 2: mixed feeding; 3: exclusive artificial feeding.

The comparative analyses of the different groups using linear regression models are listed in Table 3.

**Table 3.** Adjusted linear regression model for comparative analysis of mean differences in Z-scores for weight, length and head circumference between discharge and birth for the different study groups

Z-Score/Multiple comparisons	ΔZ-Score (discharge–birth)	P value	95%CI	
			Lower	Upper
Weight				
1–2	−0.16	0.05	−0.33	0.001
1–3	−0.10	0.19	−0.26	0.05
2–3	0.06	0.22	−0.04	0.16
Length				
1–2	0.07	0.73	−0.33	0.47
1–3	−0.27	0.17	−0.65	0.11
2–3	−0.34	0.01*	−0.58	−0.10
Head circumference				
1–2	−0.01	0.97	−0.46	0.44
1–3	−0.21	0.33	−0.63	0.21
2–3	−0.20	0.15	−0.47	0.07

Study groups: 1, exclusive breast milk; 2, mixed feeding; 3, exclusive artificial feeding. ΔZ-Score: mean difference in Z-score for weight, length, and head circumference between discharge and birth. Linear regression model adjusted for bronchopulmonary dysplasia, periintraventricular hemorrhage, SNAPPE II, weight and gestational age at birth, occurrence of enterocolitis and sepsis. *P<0.05.

No significant difference in weight or head circumference was observed between the infants studied regardless of the type of feeding they were receiving at discharge. Only length [Δ Z-score −0.34 (P-value=0.01; 95%CI=-0.58 to −0.10)] was impaired in group 2 compared to group 3, although without clinical significance.

## Discussion

Anthropometric measurements such as weight, length and head circumference are parameters used in daily clinical practice for both prenatal and postnatal nutritional evaluation.

It is known that insufficient postnatal growth of very low birth weight premature babies may result from complex interactions between genetic and environmental factors and not simply from an inadequate nutritional supply. Growth might also be affected by morbidities, endocrinological abnormalities, and administration of medications that might interfere with nutritional requirements and nutrient metabolism (14).

The nutritional goal of preterm infants in postnatal care is to achieve a growth rate that approximates the intrauterine growth and weight gain of a normal fetus of the same gestational age, without producing nutritional deficiencies, metabolic effects, toxicities or exaggerated nutritional supply (15,16).

Another concern regarding the nutrition of premature babies is that postnatal growth restriction may contribute to lower growth after discharge, and other unfavorable outcomes (14).

The nutritional evaluation of the premature babies in this study did not reveal significant differences in Z-score for weight, length or head circumference between the different groups, even after adjustment for confounding variables.

Comparative analysis of the anthropometric measurements between the different study groups did not reveal significant differences in the variables at discharge from the hospital. Only length was found to be slightly more impaired at discharge in the group of infants receiving mixed feeding compared to those exclusively receiving formula. It should be considered, however, that length is difficult to measure with accuracy in neonates.

A study conducted in the United States from 1996 to 1998 on infants with a gestational age at birth of less than 33 weeks analyzed the growth and development to 1 year of corrected age of babies fed human milk or a milk formula. Growth was found to be inversely proportional to the consumption of human milk. However, assessment of neurological development revealed that infants fed maternal milk showed a better performance (17).

Other studies that compared breastfed neonates and neonates receiving a milk formula also showed a better weight gain among those fed a formula, although without any beneficial effect on neurological development (18–20).

In a review published in 2014, British investigators who analyzed 9 trials comparing the risks and benefits of feeding preterm low birth weight babies with maternal milk from donors or with infant milk formula observed greater weight gain, length, and head circumference in the group of infants receiving formula during hospitalization. However, the risk of occurrence of necrotizing enterocolitis was higher in this group of infants (4).

Other studies did not observe greater weight and length gain among premature babies receiving a formula, with results similar to those detected in the present study. A review study conducted on 400 preterm babies with a gestational age ≤30 weeks observed a lower prevalence of necrotizing enterocolitis and retinopathy of prematurity, with no significant differences in weight gain in infants fed maternal milk compared to infants receiving a formula (21). Cristofalo et al. (22) studied 1979 premature infants with a mean gestational age of 27 weeks and observed no significant differences in weight-height gain or head circumference growth between infants fed maternal milk and infants receiving a formula.

A recently published study of newborns with less than 30 weeks of gestation and with birth weight <1250 g who were followed up for 7 years revealed that preterm infants predominantly breastfed during the neonatal period showed higher scores in tests for the evaluation of neuromotor development than infants receiving milk formulas (23). Another study of very low birth weight preterm infants also observed better cognitive and motor development in children fed maternal milk than in those receiving formulas (24).

Despite divergent results regarding weight-length gain, several studies have shown clinical benefits among infants fed maternal milk (4,17,21,23,24).

Feeding premature babies with human milk, regardless of the weight gain, offers many advantages for the health of these patients. During hospitalization, human milk feeding is related to less occurrence of necrotizing enterocolitis, sepsis and urinary tract infection, decreased gastric pH, increased gastrointestinal motility, accelerated mucosal immunity, improved gut microflora, and decreased mucosal permeability leading to reduced bacterial translocation. The benefits of human milk remain after discharge as they improve indexes of neurodevelopment that persists into adolescence, avoiding obesity, precocious puberty and other problems (25–28).

In the present study, no nutritional difference was observed at discharge between very low birth weight preterm infants fed different types of diets.

Today, despite the benefits reported in all studies, the incidence and duration of the use of maternal milk by preterm babies are usually lower than recommended. Among other factors, the lack of maternal milk use in the diet offered to these babies during hospitalization favors weaning. Common reasons for the lack of use of human milk is the anxiety of the health team regarding the rate of weight gain, although the use of an infant formula in the present study did not show nutritional advantages compared to maternal milk at the time of discharge. Another reason is the long time between birth and the transition to oral feeding, with the need for much stimulation and involvement of the health team to maintain lactation during the period of hospitalization of premature babies (29,30).

In the present study, the anthropometric measurement was made by the nursing team, being subject to error, which was a limitation. In addition, the retrospective nature of the study was another limitation, since the proportion of each type of milk offered during hospitalization for the included patients was not known. It is important to note that even patients who had a complete transition to artificial feeding also received human milk for some period during hospitalization. Therefore, we could not be sure that the group that was exclusively breastfed at the time of discharge received a greater proportion of human milk during hospitalization than the other groups.

Another important point to highlight is that group 1 consisted of a smaller number of patients (10.1% of the total), which could have biased the results, suggesting that further studies are necessary to confirm these findings.

The nutritional status of very low birth weight premature infants at discharge from the hospital was not influenced by the type of feeding offered during hospitalization. In view of the countless nutritional and immunological advantages of breast milk regarding neurodevelopment, we suggest that premature babies be exclusively fed maternal milk within neonatal units whenever possible.
